# Integrative Genomics Analysis Unravels Tissue-Specific Pathways, Networks, and Key Regulators of Blood Pressure Regulation

**DOI:** 10.3389/fcvm.2019.00021

**Published:** 2019-03-12

**Authors:** Yuqi Zhao, Montgomery Blencowe, Xingyi Shi, Le Shu, Candace Levian, In Sook Ahn, Stuart K. Kim, Tianxiao Huan, Daniel Levy, Xia Yang

**Affiliations:** ^1^Department of Integrative Biology and Physiology, University of California, Los Angeles, Los Angeles, CA, United States; ^2^Department of Genetics, Department of Developmental Biology, Stanford University Medical Center, Stanford, CA, United States; ^3^The National Heart Lung and Blood Institute's Framingham Heart Study, Framingham, MA, United States; ^4^The Population Sciences Branch and the Division of Intramural Research, National Heart, Lung and Blood Institute, Bethesda, MD, United States

**Keywords:** blood pressure, genome wide association studies, integrative genomics, regulatory networks, key drivers

## Abstract

Blood pressure (BP) is a highly heritable trait and a major cardiovascular disease risk factor. Genome wide association studies (GWAS) have implicated a number of susceptibility loci for systolic (SBP) and diastolic (DBP) blood pressure. However, a large portion of the heritability cannot be explained by the top GWAS loci and a comprehensive understanding of the underlying molecular mechanisms is still lacking. Here, we utilized an integrative genomics approach that leveraged multiple genetic and genomic datasets including (a) GWAS for SBP and DBP from the International Consortium for Blood Pressure (ICBP), (b) expression quantitative trait loci (eQTLs) from genetics of gene expression studies of human tissues related to BP, (c) knowledge-driven biological pathways, and (d) data-driven tissue-specific regulatory gene networks. Integration of these multidimensional datasets revealed tens of pathways and gene subnetworks in vascular tissues, liver, adipose, blood, and brain functionally associated with DBP and SBP. Diverse processes such as platelet production, insulin secretion/signaling, protein catabolism, cell adhesion and junction, immune and inflammation, and cardiac/smooth muscle contraction, were shared between DBP and SBP. Furthermore, “Wnt signaling” and “mammalian target of rapamycin (mTOR) signaling” pathways were found to be unique to SBP, while “cytokine network”, and “tryptophan catabolism” to DBP. Incorporation of gene regulatory networks in our analysis informed on key regulator genes that orchestrate tissue-specific subnetworks of genes whose variants together explain ~20% of BP heritability. Our results shed light on the complex mechanisms underlying BP regulation and highlight potential novel targets and pathways for hypertension and cardiovascular diseases.

## Introduction

Hypertension or elevated blood pressure (BP) is among the most prevalent, treatable risk factors for coronary artery disease (CAD), heart failure, and stroke. Systolic (SBP) and diastolic blood pressure (DBP) traits are highly heritable, with the total heritability for European/African ancestry individuals estimated to be 20/27% and 39/50% for SBP and DBP, respectively (1). Large-scale GWAS have successfully established ~800 genetic loci for SBP, DBP, and hypertension in multiple ethnic groups (2) and the number will continue to rise as the sample size increases. Some of the loci contain genes previously known or suspected to regulate BP (such as *ADM* and *NPPA*) and the remaining loci are novel.

Despite the successful identification of novel genetic loci associated with BP regulation through GWAS, pinpointing the causal genes and underlying mechanisms mediating the effects of these loci is not straightforward (3). Integration of genetic information with functional data, such as genetic variants associated with altered gene expression, or expression quantitative trait loci (eQTLs), is of critical importance to pinpoint the causal genes and their associated pathogenic mechanisms (4). Another critical gap is that the known ~800 GWAS BP loci with significance together only explains a small fraction of total BP heritability (2), a phenomenon called the missing heritability or the dark matter. Recent evidence suggests that the missing heritability can be explained by numerous additional genetic loci with moderate or subtle effects that are well under the genome-wide significance level in GWAS as well as the interactions between multiple genetic loci (5). Such multigenic interactions can be captured using gene regulatory networks, and our recent modeling of blood pressure networks using whole blood transcriptome data has highlighted the importance of inflammatory pathways in blood pressure regulation (6). However, BP regulation most likely involves many more genes functioning in numerous biological processes in diverse tissues such as kidney, heart, liver, and the vasculature (2, 7). Systematic modeling of multidimensional omics data that capture tissue-specific blood pressure networks informed by genetic loci of strong, moderate, to subtle effects will likely provide a more comprehensive understanding of BP mechanisms.

Here we employed an integrative genomics strategy leveraging multiple genetic and genomic datasets. Previous applications of this strategy have successfully identified novel mechanisms of CAD and other complex diseases (4, 8, 9). Once integrated, these multidimensional datasets have the potential to delineate the genes, pathways, and epistatic interaction subnetworks associated with BP that are informed by genetic signals with a wide range of effect sizes.

## Methods

### Overall Analysis Design

**Figure 1:**
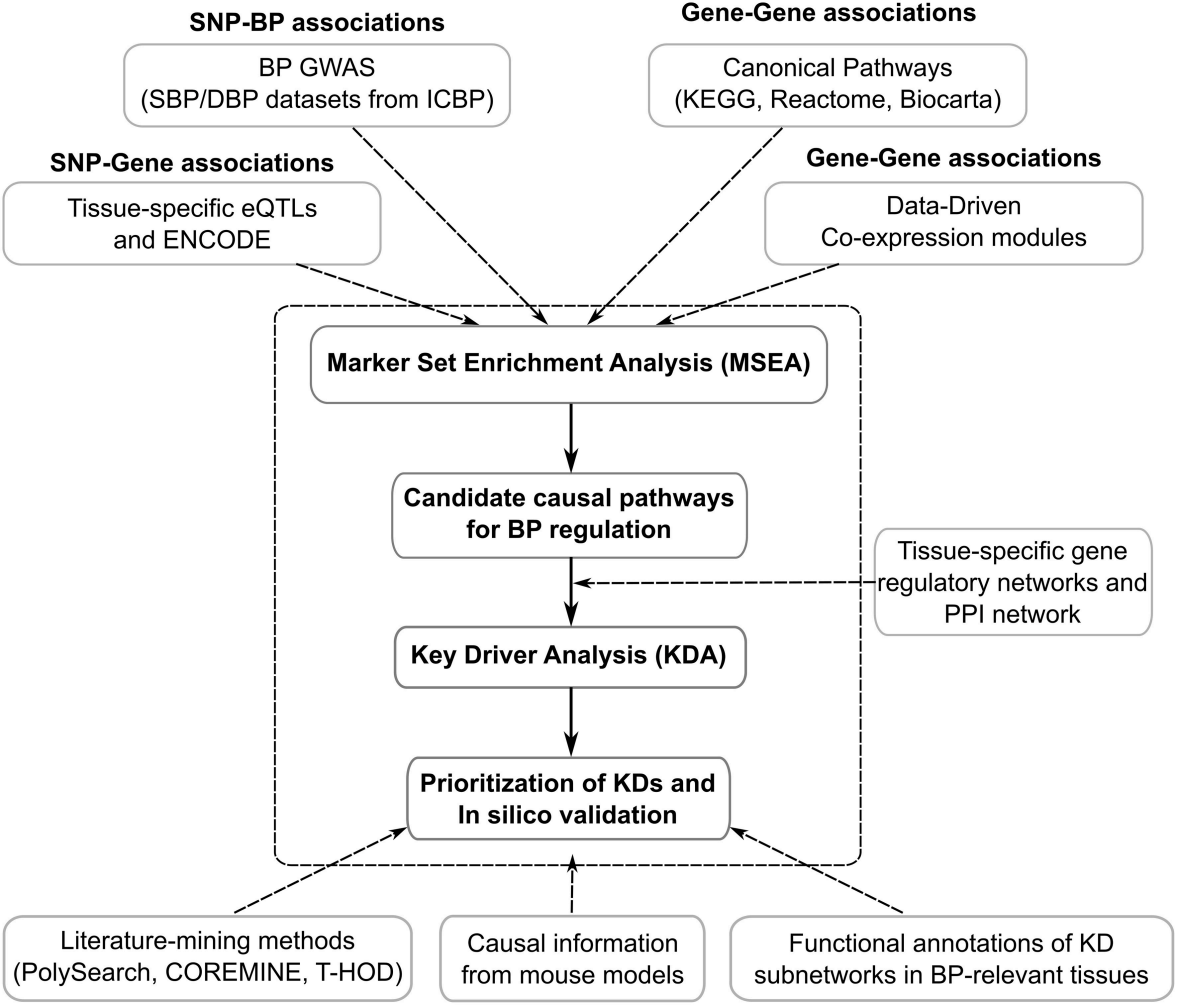
The integrative genomics framework for identifying genetically informed biological processes and networks and for prioritizing key drivers of BP regulation.

Figure 1 shows the general flowchart of the study. First, we utilized the human genetic association data (i.e., GWAS) from the ICBP, which provides the full spectrum of statistical associations between SNPs and clinically measured SBP and DBP (not limiting to the top significant loci). Second, we curated eQTLs (SNPs under eQTLs are defined as eSNPs) from diverse tissues, which have been confirmed to be enriched for complex disease loci (10) and provide functional support for tissue-specific connections between SNPs and genes in a data-driven manner. To further enrich functional annotation, we incorporated information from the Encyclopedia of DNA Elements (ENCODE) studies (11). Third, to provide a holistic view of the organization of genes and reveal the most important regulatory hubs in a given tissue, we included knowledge-based metabolic and signaling pathways and data-driven gene networks from various tissues to improve the detection of multigenic disease processes. The general analytical pipeline, Mergeomics, has been implemented as an open-access web-server (http://mergeomics.research.idre.ucla.edu/) (12) as well as an open-access R Bioconductor package (https://bioconductor.org/packages/release/bioc/html/Mergeomics.html) (13). Ethical standards and procedures were followed throughout the study.

### GWAS of SBP, DBP, Hypertension, and CAD

The summary statistics of GWAS for SBP, DBP, and hypertension was obtained from the ICBP, which was formed by two consortia, the CHARGE-BP consortium (Cohorts for Heart and Aging Research in Genomic Epidemiology—blood pressure) and the GBPGEN consortium (Global Blood Pressure Genetics Consortium) (14). (dbGaP accession: phs000585.v2.p1). The study is comprised of 200,000 individuals of European descent in a multi-stage design from 29 studies. More than 906,600 SNPs were genotyped using Affymetrix Genome-Wide Human SNP Array 6.0. Imputation was further carried out to obtain information for up to 2.6 million SNPs using the HapMap CEU (Utah residents with ancestry from northern and western Europe) panel. SNPs with minor allele frequency (MAF) <1% were removed. Finally, a total of ~2.5 million SNPs tested for association with systolic and diastolic blood pressure were used in our study. The 1,000 Genomes-based CAD GWAS dataset was retrieved from CARDIoGRAMplusC4D Consortium (http://www.cardiogramplusc4d.org/media/cardiogramplusc4d-consortium/data-downloads/cad.additive.Oct2015.pub.zip) (15). The hypertension and CAD GWAS were used to connect the SBP/DBP related findings to disease conditions. All statistical association *p*-values for all SNPs, regardless of significance level, were used in our downstream analysis.

### Mapping SNPs to Genes and Removal of SNPs in Linkage Disequilibrium (LD)

We used different mapping methods that are based on (a) chromosomal distance, (b) eQTLs, or (c) ENCODE to link GWAS SNPs to their potential target genes.

- We used a standard distance-based approach where a SNP was mapped to a gene if within 50 kb of the respective gene region.
- The expression levels of genes can be seen also as quantitative traits in GWAS. Hence, it is possible to determine eQTLs and the expression SNPs (eSNPs) within the eQTLs that provide a functionally motivated mapping from SNPs to genes. Moreover, the eSNPs within the eQTL are specific to the tissue where the gene expression was measured and can therefore provide mechanistic clues regarding the tissue of action when intersected with BP-associated SNPs. Results from eQTL studies in human adipose tissue, artery, liver, brain, and blood (16– 28) were combined with the eQTLs from the same tissues in the GTEx database (29). In addition, we included eQTLs from 44 additional tissues from studies with smaller sample sizes including the GTEx (30) (https://storage.googleapis.com/gtex_analysis_v7/single_tissue_eqtl_data/GTEx_Analysis_v7_eQTL_all_associations.tar.gz) and eQTLs in kidney at FDR <5% (Kim et al., unpublished data) but the statistical power of these eQTL sets was limited. We included both *cis*-eSNPs (within 1 Mb distance from gene region) and *trans*-eSNPs (beyond 1 Mb from gene region), at a false discovery rate (FDR) <5%. However, for these tissues, the number of eSNPs after LD trimming was small and lacked power to detect BP-associated signals. To improve statistical power, we included eSNPs at *P* < 1.0E-5 from these 44 tissues as “suggestive” eQTL sets.
- In addition to eQTLs and distance-based SNP-gene mapping approaches, we integrated functional data sets from the Regulome database (11) which annotates SNPs in regulatory elements in the *Homo sapiens* genome based on the results from the ENCODE studies (31).

Using the above mapping approaches, the following sets of SNP-gene mappings: eSNP adipose, eSNP artery, eSNP liver, eSNP blood, eSNP brain, eSNP all (i.e., combing all the tissue-specific eSNPs above), Distance (chromosomal distance-based mapping), Regulome (ENCODE-based mapping), Combined (combing all the above methods), and 44 suggestive eQTL sets.

We observed a high degree of LD in the eQTL, Regulome, and distance-based SNPs, and this LD structure may cause artifacts and biases in the downstream analysis. For this reason, we devised an algorithm to remove SNPs in LD while preferentially keeping those with a strong statistical association with SBP/DBP. We chose a LD cutoff (*r*^2^ < 0.7) to remove redundant SNPs in high LD.

### Knowledge-Based Biological Pathways

We included 1,827 canonical pathways from the Reactome (Version 45), Biocarta, and the Kyoto Encyclopedia of Genes and Genomes (KEGG) databases (32, 33). In addition to the curated pathways, we constructed two positive control BP pathways based on known BP loci (*p* < 1.0E-5) and candidate genes from the GWAS Catalog (GWAS *p* < 5.0E-8) (34) for SBP and DBP separately. We also curated hypertension/CAD positive control gene sets based on GWAS Catalog (*p* < 1.0E-5). In addition, the CAD positive control genes were complemented with the CADgene V2.0 database, which contains 583 CAD related genes and detailed CAD association information from about 5,000 publications. These gene sets serve as positive controls to validate our computational method.

### Data-Driven Modules of Co-expressed Genes

Beside the canonical pathways, we used co-expression modules that were derived from a collection of genomics studies of liver, adipose tissue, aortic endothelial cells, brain, blood, kidney, and muscle (GEO accession numbers: GSE7965, GSE25506, GSE9588, GSE24335, GSE20142, GSE20332, GSE22070, GSE2814, GSE3086, GSE2814, GSE3086, GSE3087, and GSE3088, and GSE30169) (16–19, 21, 22, 35–38). For each dataset, we extracted the normalized gene expression profile and reconstructed co-expression networks using the established WGCNA R package (39). Modules with size smaller than 10 genes were excluded to avoid statistical artifacts, yielding a total of 2,705 co-expression modules in this study. We included these tissue-specific co-expression networks to confirm whether known tissue types for BP could be objectively detected and whether any additional tissue types are also important for BP regulation.

These data-driven modules along with the knowledge-driven pathways in the previous section were used together to capture gene sets containing functionally related genes in a wide variety of tissue and functional settings.

### Marker Set Enrichment Analysis (MSEA)

We applied MSEA (13) to identify pathways/co-expression modules that demonstrate enrichment for genetic association with SBP, DBP, hypertension, or CAD using the same parameters. MSEA employs a chi-square like statistic with multiple quantile thresholds to assess whether a pathway or co-expression module shows enrichment of disease SNPs compared to random chance based on the full spectrum of association statistics for each GWAS dataset. For each pathway or co-expression module, 10,000 permuted gene sets were generated, and enrichment *P*-values were determined from a Gaussian distribution approximated using the enrichment statistics from the permutations as detailed in Shu et al. (13). Benjamini-Hochberg FDR was estimated across all pathways and modules tested for each GWAS. Pathways or co-expression modules with FDR < 5% in at least one SNP-gene mapping were considered statistically significant.

### Construction of Independent Supersets and Confirmation of BP Association

Because the significant pathways or co-expression modules were from multiple sources, there were overlapping or nested structures among the gene sets. To make the results more meaningful, we constructed independent supersets that captured the core genes from groups of redundant pathways and co-expression modules (Figure 1). We merged the 42 common pathways associated with SBP/DBP using a merging algorithm in Mergeomics (13). After merging, we annotated each superset based on function enrichment analysis of the known pathways from the Gene Ontology and KEGG databases (Bonferroni-corrected *P* < 0.05 in Fisher's exact test). The supersets were given a second round of MSEA to confirm their significant association with BP using Bonferroni corrected *P* < 0.05 as the cutoff.

### Key Driver Analysis (KDA)

We used a KDA algorithm (40) to identify potential key driver (KD) genes of the BP-associated supersets. KDA overlays BP-associated gene sets that were discovered by MSEA onto graphical network models detailing molecular interactions among genes to see if a particular subnetwork was significantly enriched for disease genes, using a chi-square like statistic analogous to the one used for MSEA. Statistical significance of KDs was estimated by permuting the gene labels in the network for 10,000 times and estimating the *P*-value based on the null distribution. To control for multiple testing, stringent adjustment (FDR < 1%) was used to focus on the top robust KDs. Graphical networks used sources including tissue-specific Bayesian regulatory networks available for seven tissues (including cardiac muscle, artery, adipose, blood, liver, brain, and kidney, described previously (7) and a protein-protein interaction network from Human Protein Reference Database (HPRD) database (41).

To cross-validate the top ranked KDs *in silico*, we used literature-mining methods (PolySearch, COREMINE, and T-HOD), searched mouse phenome database (http://www.informatics.jax.org/), and examined their association with BP in the latest GWAS to assess supporting evidence for their role in BP regulation. We also retrieved gene essentiality information (42) from an exome sequencing study of 60,706 humans (Supplementary Table 5) (42) to evaluate the biological importance of the predicted KDs. This study considered genes with a probability of being loss-of-function (LoF) intolerant >= 0.9 to be essential genes, where counts of the observed and expected variants were utilized to predict whether a given gene is significantly intolerant to LoF. Genes were classified into three groups within the context of tolerance to LoF, null/complete toleration, recessive/heterozygous toleration, and haploinsufficient/intolerant.

### Heritability Estimates of GWAS Hits and KD Subnetworks

We used the Heritability Estimator from Summary Statistics (HESS) (43) to estimate the total genome-wide SNP heritability (*h*^2^) as well as the portion explained by the KD subnetworks of SBP/DBP. We define GWAS hits as SNPs with *p* < 5.0E-8. To correct for any potential bias in the large numbers of KD subnetwork genes for SBP/DBP, we implemented a permutation strategy by generating 10,000 random gene sets of matching sizes for the top KD subnetworks of SBP/DBP to derive permutation-based significant *P*-values for the heritability estimates, where *P*_*KD*_ = (number of $h_{g,random}^{2}$ > $h_{g,KD}^{2}$)/10000. The reference heritability of SBP/DBP was reported by a recent study (44), which studied 2,889 twin pairs not on any BP-lowering therapy from the Twins UK cohort, and estimated the additive genetic variance for baseline BP, long-term average BP, BP trajectory (rate of change of BP in mmHg/year) and BP variability (coefficient of variation and average real variability over an $h_{g}^{2}$ average of 3.2 visits).

## Results

### Identification of BP-Associated Pathways and Co-expression Network Modules

Out of the 4,532 gene sets (1,827 canonical pathways and 2,705 data-driven co-expression modules), we identified 96 and 78 that were significantly associated with SBP and DBP, respectively, in at least one SNP-to-gene mapping approach (FDR < 5%; Supplementary Table 1). As expected, the predefined positive controls based on GWAS catalog for SBP and DBP were among the top signals for their corresponding traits.

Among the significant signals, 42 gene sets were shared between SBP and DBP (Table 1). These included the previously documented hypertension processes, such as angiotensin II (45), cell-cell junction organization (46), and muscle contraction (47). Other plausible pathways included NOTCH signaling, platelet production, insulin secretion/signaling, immune and inflammation, corticosteroids, and cell cycle pathways (Table 1). Among the gene sets unique to either SBP or DBP were “Wnt signaling” and “mammalian target of rapamycin (mTOR) signaling” for SBP, and “cytokine network” and “tryptophan catabolism” for DBP (Supplementary Table 1).

**Table 1:** Shared pathways or co-expression modules between SBP and DBP.

**Gene set ID**	**Descriptions**	**Informative eQTLs/mapping*^†^***	**Supersets**	**Top KDs**
SBP Positive	Positive controls for SBP	1,2,3,4,5,6,7,8,9	S1: positive controls (197)	*PON1, PTK2, HIPK2, SPTBN1, IRS1*
DBP Positive	Positive controls for DBP	1,2,3,4,5,6,7,8,9		
rctm1133	Signaling by NOTCH	1,5,6,7,8,9	S2: NOTCH signaling (122)	*NOTCH2, NOTCH1, RBPJ, HDAC3, NOTCH3*
rctm0893	Pre-NOTCH expression and processing	1,3,5,6,7,9		
Co:4279	(Protein modification, catabolism)^*^	1,6,8,9	S3: protein catabolism (129)	*PYGL, NAMPT, SLC2A3, ZFP36, AQP9*
Co:4964	(Protein modification, catabolism)^*^	1,6,8,9		
Co:4610	(Carbohydrate metabolism)^*^	1,3,4,6,8,9		
M2164	Leukocyte transendothelial migration	1,4,5,6,8,9	S4: cell adhesion and junction (219)	*MSN, FYN, RAC2, ARHGDIB, LCK*
rctm1276	Tight junction interactions	1,5,6,7,8,9		
rctm0225	Cell-cell junction organization	1,5,6,7,9		
M16476	Cell adhesion molecules (CAMs)	1,5,6,8,9		
M8728	Hypertrophic cardiomyopathy (HCM)	1,7,8,9	S5: cardiac muscle contraction (146)	*COX5B, COX4I1, NDUFS3, COX7B, COX6B1*
M17673	Cardiac muscle contraction	1,3,4,5		
M835	Dilated cardiomyopathy	1,3,5,6,7,8,9		
rctm0731	Muscle contraction	1,3,4,5,6,7,8,9	S6: smooth muscle contraction (49)	*CALD1, TTN, ACTA1, MYLPF, KBTBD10*
rctm1162	Smooth muscle contraction	1,4,6,7,8,9		
M3896	Inositol phosphate metabolism	1,3,6,7,8,9	S7: phosphatidylinositol signaling (82)	*BTK, TRPC3, KIT, EGFR, GRM5*
M9052	Phosphatidylinositol signaling system	1,3,6,7,8,9		
rctm0996	Regulation of Insulin Secretion	1,3,6,7,8,9	S8: insulin secretion (199)	*GNB1, PRKACA, GNAS, VIPR1, GNB2*
rctm0449	G alpha (s) signaling events	6,8,9		
rctm0598	Integration of energy metabolism	7,8,9		
M18155	Insulin signaling pathway	1,6,8,9	I1: insulin signaling (137)	*GRB2, HRAS, INSR, AKT1, IRS1*
M16004	Antigen processing and presentation	1,2,3,5,6,7,8,9	S9: immune and inflammation (398)	*HLA-C, ZC3H7B, PPP3R1, MIDN, CREBL1*
M917	Complement pathway	1,3,6,8,9		
Co:4555	(Adaptive immune system)^*^	1,3,5,6,7,8,9		
Co:black	(Immune system)^*^	1,5,6,7,9		
M3342	Integrin signaling pathway	1,3,6,7,8,9	S10: Integrin Signaling (111)	*FN1, COL4A1, ITGB3, PTK2, PXN*
rctm0602	Integrin cell surface interactions	1,6,7,8,9		
Co:4818	(Gene expression)^*^	1,4,5,6,7,8,9	S11: Gene Expression (313)	*PPP3R1, CCBL2, ZC3H7B, MAFG, MIDN*
Co:4261	(Gene expression)^*^	1,6,7,8,9		
Co:4603	(Gene expression)^*^	1,6,7,8,9		
rctm1391	mRNA Decay by 5′ to 3′ Exoribonuclease	6,7,8,9		
Co:cyan	(RNA processing)^*^	7,8,9		
rctm0622	Intrinsic pathway for apoptosis	2,3,6,7,8,9	S12: Cell Cycle (109)	*HIST1H2BC, HIST1H2BM, HIST1H2BK, MDM4, AKT1*
rctm0676	Meiotic Recombination	1,6,7,8,9		
M10145	PTEN dependent cell cycle arrest and apoptosis	7,8,9		
M14899	Angiotensin II mediated activation of JNK Pathway via Pyk2 dependent signaling	1,3,6,7,8,9	I2: Angiotensin II-induced JNK activation (36)	*PIK3R1, RAF1, EGFR, MAPK14, MAPK1*
rctm0411	Factors involved in megakaryocyte development and platelet production	7,8,9	I3: Platelet Production (125)	*EP300, AURKA, PML, KIF2C, AKAP1*
M6382	Regulation of autophagy	1,6,7,8,9	I4: Autophagy (35)	*MAP1LC3B, GABARAP, GABARAPL2, IFNAR2*
M17400	ALK in cardiac myocytes	7,8,9	I5: ALK in Cardiac Myocytes (37)	*BMPR1A, TGFBR1, BMP2, ENG, TGFBR2*
M10066	Corticosteroids and cardioprotection	7,8,9	I6: Corticosteroids and Cardioprotection (20)	*ESR1, HSP90AA1, NR3C1, INSR, AR*
Co:5504	(Small molecule metabolic process)^*^	1,3,4,6,7,8,9	I7: Small Molecule Metabolism (114)	*AGPAT2, MECR, AKR1C3, GPX4, FAH*

* *The coexpression modules were annotated using MSigDB pathways (www.broadinstitute.org/msigdb) with FDR < 5%*.

† *Number 1 to 9 represent: Adipose eSNP (1), Artery eSNP (2), Blood eSNP (3), Brain eSNP (4), Liver eSNP (5), all eSNP (6), Distance (7), Regulome (8), and Combined (9), respectively*.

The use of tissue-specific eQTLs allowed us to implicate the potential tissues where the BP-associated processes may function (Supplementary Table 1). Out of the 44 tissue-specific eQTL sets, those from the adipose, liver, blood, and brain tissues appeared to be more informative, although this could be due to the higher statistical power resulting from the abundance of eQTLs from these well-studied tissues. Adipose eQTLs were shown to be informative for the majority of the pathways or co-expression modules identified, and insulin secretion/signaling, autophagy, cell cycle and protein modification processes were mainly identified when adipose eQTLs were used. Use of smaller eQTLs datasets such as those from heart and kidney tissues, which were previously implicated in BP (7), did not yield significant BP-associated pathways. This is likely due to the limited statistical power instead of lacking biological relevance. Indeed, by lowering the stringency of eQTLs from FDR < 5% to *P* < 1.0E-5 to include more eQTLs from these tissues, we found suggestive pathways (e.g., response to wounding, cell-cell adhesion, and G-protein coupled receptor signaling) informed by eQTLs from artery, heart, pancreas, and testis tissues (FDR < 5%; Supplementary Table 2).

### Construction of Supersets for SBP/DBP and Functional Annotation

We next focused on the 42 significant gene sets (pathways/co-expression modules) shared between SBP and DBP as they reflect reproducible signals for BP regulation. To minimize redundancy in the biological processes captured in these gene sets, we merged 35 overlapping gene sets into 12 independent supersets and kept the other 7 non-overlapping gene sets intact (Table 1). The resulting 19 non-overlapping gene sets represent a diverse range of molecular pathways, including NOTCH signaling, protein catabolism, cell adhesion and junction, cardiac and smooth muscle contraction, phosphatidylinositol signaling, insulin secretion/signaling, immune/inflammation, integrin signaling, gene expression regulation, cell cycle, angiotensin II-induced JNK activation, platelet, autophagy, and corticosteroids and cardioprotection. We confirmed that these merged supersets still captured the BP-relevance demonstrated by their subcomponents by performing a second round of MSEA (Supplementary Table 3).

### Identification and Prioritization of key Regulators in the BP-Associated Supersets

To identify and prioritize the central regulatory components (termed as key drivers, KD) among the large number of genes in the BP supersets, we performed a key driver analysis (KDA; details in Methods) (13) on the 19 supersets shared between SBP and DBP using 8 graphical networks including 7 tissue-specific Bayesian networks [cardiac muscle, artery, adipose, blood, liver, brain, and kidney, as these tissues have been implicated in BP by previous (7) and our studies] and a PPI network. For each superset in each network, we focused on the top five ranked KDs satisfying FDR < 1%, yielding 295 unique KD genes, among which 29 were shared by >=2 supersets and 6 (*COX5B, FN1, COL4A2, COX4I1, NDUFS3*, and *HLA-C*) were shared in >=3 networks (Supplementary Table 4). The low number of shared KDs for the BP-associated gene sets between tissue-specific networks suggests tissue-specific regulation of BP genes and pathways.

We cross-checked these top KD genes for their role in BP and functional significance through a comprehensive *in silico* analysis using multiple literature mining tools, databases of gene knockout and mutation mouse models, and recent GWAS findings (Supplementary Table 5). Our search revealed both known KDs showing connections to hypertension or relevant conditions in one or more types of studies (99 KDs; such as *GNAS, SLC2A3, IRS1, ADM*, and *SERPINE1*) and relatively novel KDs in BP studies (such as *SPTBN1* and *GNB1*). Moreover, the top KDs were significantly enriched with essential human genes (41) (Supplementary Table 5; Fisher Exact test *P* = 5.12e-7).

Additionally, a number of suggestive genes arose from the vascular tissue data, most notably those implicated in extracellular matrix (ECM) homeostasis and smooth muscle contraction with emphasis on angiotensin signaling. The genes highlighted as having specific effects on the ECM are *TIMP2, PSAP, FN1, VCAN*, and *LOX*. Those implicated in smooth muscle contraction include *CALD1, DUSP5*, and *TAGLN*.

### Top KD Subnetworks of BP Regulation

We retrieved the top KD subnetworks in BP-relevant tissues including cardiac muscle, artery, adipose, blood, liver, brain, and kidney, as well as in the PPI network. As shown in Supplementary Figure 1, BP-associated processes are closely orchestrated by KDs in the adipose, artery, blood, and liver subnetworks. The top KD subnetworks demonstrated high tissue specificity, for example, subnetworks involved in insulin secretion/signaling were only overrepresented in adipose and liver tissues (Supplementary Figure 1). Similar results were found for the BP subnetworks from the other tissues (the detailed interactions in the eight KD networks are listed in Supplementary Table 6).

### BP Heritability Explained by the KD Subnetworks

We estimated the total genome-wide SNP heritability (*h*^2^) and the fractions of heritability explained by the top KD subnetworks using HESS (43) based on the ICBP GWAS summary statistics. We found that while the significant GWAS SNPs (14) can only explain 4.82% and 4.46% of trait variance in SBP and DBP, respectively, which agreed with the estimates from a recent BP GWAS study (2), KDs and genes in the top KD subnetworks in the various tissues explained much higher proportions of heritability (Table 2). For example, the top 54 KDs and their subnetwork genes in the adipose tissue (Supplementary Figure 1) can explain 19.8% and 21.8% of the heritability of SBP and DBP, respectively. This increase in heritability estimates for the KD subnetworks over GWAS top hits is less likely to be driven by the number of network genes, as random subnetworks containing matching numbers of genes explained much lower proportions of heritability (*P* < 0.0001).

**Table 2:** Estimates of BP heritability explained by tissue-specific KD subnetworks.

**Traits**	$h_{g}^{2}\left(\%SE\right)$^*^	**$h_{\text{pub}}^{2}(95\%\text{CI})$^†^**	**$h_{\text{GWAS}}^{2}(\%\text{SE})$^‡^**	**Networks**	**$h_{KD\_\text{sub}}^{2}\left(\%\text{SE}\right)$^§^**	**$h_{\text{random}}^{2}\left(\%\text{SE}\right)$^||^**	***P*-values of top KD subnetworks**
SBP	45.5 (0.82)	56 (53, 59)	1.36 (0.20)	Adipose	19.76 (0.61)	5.12 (0.76)	<1.0e-4
				Artery	16.66 (0.37)	4.61 (0.82)	<1.0e-4
				Blood	18.24 (0.51)	4.82 (0.81)	<1.0e-4
				Brain	8.36 (0.35)	3.74 (0.66)	<1.0e-4
				Cardiac Muscle	13.32 (0.34)	3.95 (0.76)	<1.0e-4
				Kidney	7.65 (0.67)	3.80 (0.72)	<1.0e-4
				Liver	17.35 (0.40)	4.67 (0.65)	<1.0e-4
				PPI	22.65 (0.57)	5.10 (0.65)	<1.0e-4
DBP	62.3 (0.81)	61 (58, 64)	1.49 (0.28)	Adipose	21.75 (0.28)	7.42 (0.89)	<1.0e-4
				Artery	17.07 (0.21)	5.25 (0.76)	<1.0e-4
				Blood	23.34 (0.35)	8.37 (0.75)	<1.0e-4
				Brain	15.06 (0.24)	4.65 (0.63)	<1.0e-4
				Cardiac Muscle	15.55 (0.36)	4.45 (0.67)	<1.0e-4
				Kidney	8.90 (0.56)	4.60 (0.65)	<1.0e-4
				Liver	18.39 (0.45)	4.67 (0.83)	<1.0e-4
				PPI	24.73 (0.41)	6.26 (0.75)	<1.0e-4

* Total SNP heritability estimated by HESS;

† heritability of SBP/DBP based on reports by a recent study detailed in Methods;

‡ heritability explained by GWAS hits (P < 5.0E-8);

§ heritability explained by the subnetworks of the top KDs in each tissue network (the tissue-specific subnetworks were listed in Supplementary Table 6);

|| *average values of heritability explained by subnetworks of random genes that are not KDs; and P-values represent comparisons between top KD and random gene subnetworks*.

### Overlap of BP Pathways/Networks With Those of Hypertension and CAD

We explored the relationship between BP-associated pathways/networks with those for hypertension and CAD. Out of the 42 SBP/DBP common pathways (Table 1), five showed significant enrichment for hypertension GWAS (14) signals (FDR < 0.05), including “Antigen processing and presentation,” “Insulin signaling pathway,” and “Integration of energy metabolism,” and the two positive control sets for SBP and DBP. Six of the SBP/DBP common pathways also showed significant enrichment for CAD GWAS (15) signals, including “ALK in cardiac myocytes,” “Factors involved in megakaryocyte development and platelet production,” “Integrin cell surface interactions,” “Meiotic recombination,” and “Antigen processing and presentation”. In addition, 19 out of the top BP KDs (Supplementary Table 5; e.g., *SHC1, FN1, APOB, COL4A1, RELA*, and *ADM*) were CAD susceptibility genes based on GWAS studies (8, 48). Notably, 10 KD genes highlighted by our analysis ( e.g*., TGFBR2, LAMC1, KIF15*, and *RBPMS*) have only just been implicated as causal genes by the most recent blood pressure GWAS study (2). Moreover, many hypertension/CAD genes (Supplementary Table 7) are in the subnetworks of the top KDs (Supplementary Table 6). These molecular level overlaps support the strong mechanistic connections between BP and incident diseases.

## Discussion

While elevated BP has a significant genomic contribution, this phenotype has been historically multidimensional in its physiological induction. Previous high-throughput GWAS and transcriptome studies have revealed a plethora of genomic changes contributing to BP in different populations. However, an integrative systems analysis fully utilizing the complementarity of diverse omics data has not been conducted to capture a comprehensive, tissue-specific view of BP regulation. Due to the highly interconnected nature of the genome and molecular regulatory systems, finding the gene sets most relevant in pathogenesis through the inherent noise of each expressed gene is challenging. Based on the predictions made via the “omnigenic” model, the vast majority of heritability arises from the multitude of genes which have indirect effects on disease through their association with central regulators (49). Our previous studies on other complex diseases indeed support that GWAS genes are mostly peripheral genes in gene networks that are regulated by key driver genes (8, 9).

Here, to investigate the gene regulatory networks and pathways governing BP regulation, we integrated the full association spectrum of BP GWAS (not limited to top GWAS hits, but included moderate and subtle signals as well), functional genomics information (eQTLs and ENCODE), knowledge-driven pathways, and data-driven networks to uncover biological processes and tissue-specific networks mediating the actions of BP GWAS signals. Our study revealed a diverse set of pathways significantly associated with SBP/DBP based on genetic evidence. These pathways are connected in tissue-specific networks via central regulators, both known (e.g., *GNAS, SLC2A3*, and *IRS1*) and novel (e.g., *SPTBN1* and *GNB1*), revealing critical interactions and regulatory cascades underlying BP maintenance. The KDs and subnetworks collectively explain ~20% heritability for SBP/DBP, substantiating the much improved power of our network approach in capturing key BP genes and processes compared to conventional approaches. Furthermore, many of the BP pathways and networks were shared with CAD, thus shedding light on the mechanistic connections between CAD and its clinical risk factor hypertension.

**Figure 2:**
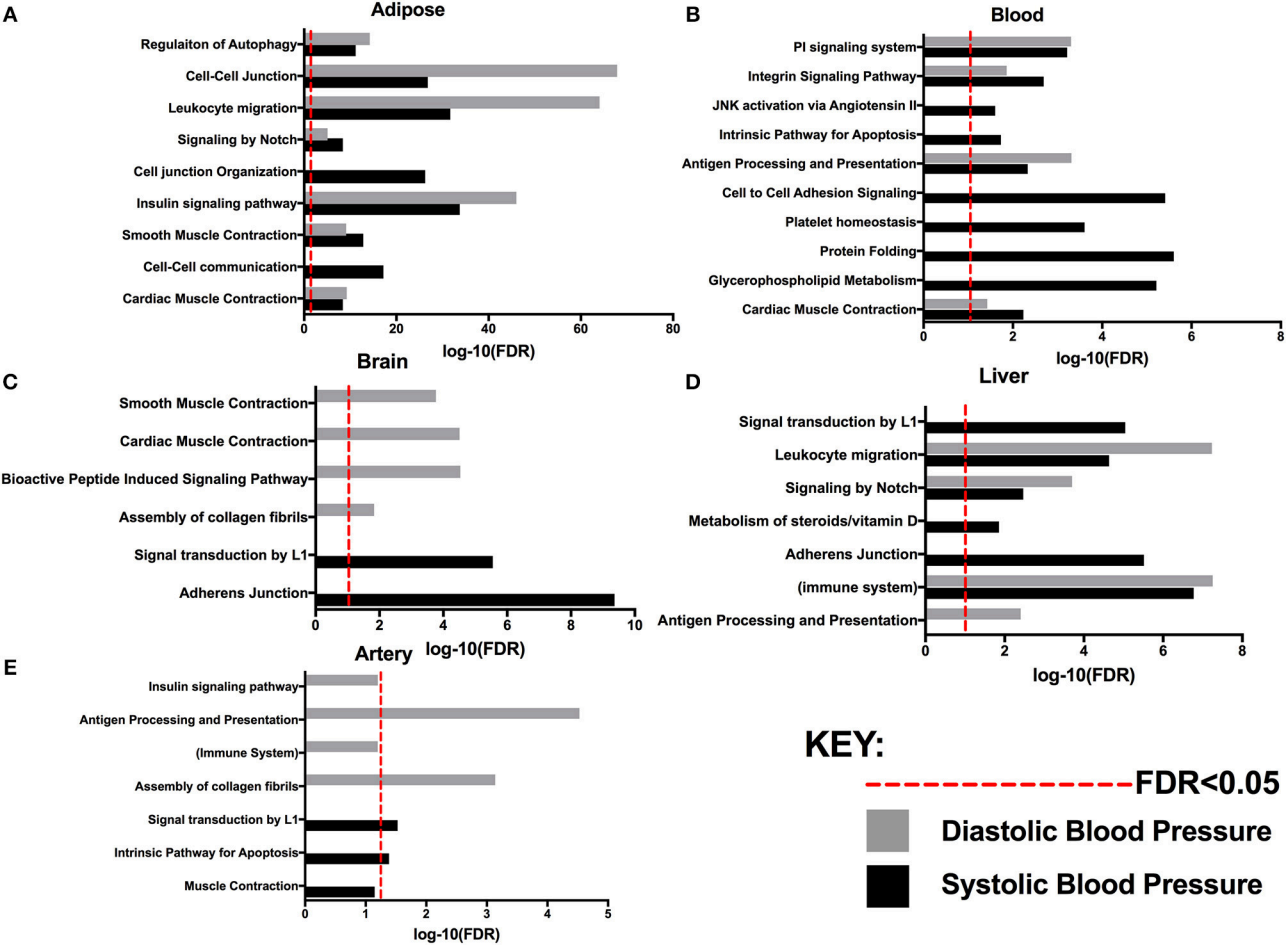
Pathways associated with systolic and/or diastolic blood pressure with FDR determined by MSEA analysis from various tissues: **(A)** Adipose. **(B)** Blood. **(C)** Brain. **(D)** Liver **(E)** Artery.

Compared to several previous integrative network studies of BP involving whole blood transcriptome and BP GWAS, (20, 33) our study incorporated eQTL and network information from a comprehensive list of tissue types that capture pathways or networks that are either systemic or tissue-specific, revealing complex, systems-level regulation of BP (summarized in Figure 2). The detected processes can be categorized into a number of general classes, such as muscle contraction, immune and inflammation, cell adhesion and junction, protein catabolism, and angiotensin pathway. Therefore, our omics-driven systems biology study depicts a much more comprehensive landscape for BP regulation that includes both known processes targeted by antihypertensive drugs (50) (angiotensin II receptor blockers targeting the angiotensin pathway) and additional processes that may lead to novel therapeutic strategies for hypertension and CAD.

The use of tissue-specific functional genomics information allowed us to detect potential tissue specificity in the BP processes identified in our study. For example, autophagy regulation and insulin signaling showed genetic association with BP traits when adipose tissue eQTLs were used (Table 1); our network models also suggest that the insulin signaling pathway in adipose tissue closely connects with other critical BP processes, such as cell-cell adhesion and cell junction organization (Figure 2, Supplementary Figure 1). The link of adipose tissue biology and insulin resistance to blood pressure homeostasis has been noted before (51, 52). Our results suggest that autophagy and insulin signaling are adipose pathways contributing to BP regulation. The connection between autophagy and insulin signaling is supported by previous evidence that adipose-specific deletion of autophagy modulators (e.g., *ATG7*) reduces white adipose mass, increases the proportion of brown adipocytes and subsequently promotes the oxidation of free fatty acids, leading to enhanced insulin sensitivity (53). A recent study unraveled that spermidine, an anti-aging molecule, reduced SBP and prevented cardiac hypertrophy and a decline in diastolic function through autophagy-related protein *ATG5* (54). Another study identified autophagy modulators as potential therapeutic targets of FDA-approved antihypertensive drugs, such as the imidazoline receptors and l-type Ca^2+^ channels (55). These lines of evidence substantiate that autophagy and insulin resistance processes in adipose may contribute to BP regulation.

Beside the common pathways between SBP and DBP discussed above, the trait-specific processes are also of value to help understand the differential regulatory mechanisms between the two traits. For instance, we found that Wnt signaling is more associated with SBP while tryptophan catabolism tends to be more involved in DBP regulation (Supplementary Table 1). In the central nervous system of spontaneously hypertensive rats, Wnt signaling regulates SBP by downregulating a glycogen synthase kinase 3β-mediated pathway to enhance insulin signaling (56). As for DBP-specific tryptophan catabolism, the central enzyme indoleamine 2,3-dioxygenase (IDO) in the pathway metabolizes the essential amino acid tryptophan to kynurenine. There was an inverse association between IDO activity and DBP but no association with SBP (57). Further investigations of these trait-specific pathways are warranted.

In addition to retrieving BP-relevant processes and pathways in a tissue-specific fashion, our network modeling detected potential KDs of these processes. These KDs include both known BP genes (e.g., *GNAS, SLC2A3*, and *IRS1*) and novel genes (e.g., *SPTBN1* and *GNB1*). The KD *SPTBN1* in “Positive Control,” is associated with multiple BP GWAS susceptibility genes (including *CPEB4, FBXL19, NPR3*, and *HIPK2*) in the adipose network (Supplementary Figure 1). It encodes βII-spectrin, which has been proven to play an essential role in the regulation of bone mineral density (58). On the other hand, bone mineral density was significantly lower in hypertensive subjects compared with normotensive subjects and was inversely correlated with SBP (59). Another novel KD *GNB1* in “Insulin regulation” encodes a G protein β subunit, multiple mutations of which were found to affect the protein interface that binds Gα subunits (*GNAS*), which has been genetically and clinically associated with hypertension (60). These lines of evidence support the potential importance of the novel KDs in BP regulation.

Several interesting observations emerged from relationship between the top KDs we found and BP GWAS signals. First, we observed a lack of GWAS signals in the predicted KDs for direct genetic association with BP, with only 22 (e.g., *PTPN11, INSR, SLC2A4*, and *GNAS*) out of the 295 predicted KDs to be candidate BP GWAS genes (2, 61). This may be a result of negative selection pressure because of the critical roles of the KDs in tissue-specific networks. In support of this hypothesis, the top KDs we found were significantly overrepresented within biologically essential genes which demonstrates few protein-truncating variants due to the critical nature of their functions (42). This helps explain why KDs are commonly missed in GWAS and the power of our network approach in uncovering potential missing disease genes and processes. The lack of KD signals in GWAS does not undermine their critical regulatory role in disease processes, as the KD subnetworks collectively explain significantly more heritability than the subnetworks of random genes as well the top BP GWAS loci for both SBP and DBP (Table 2). These results substantiate the improved ability of network approach in capturing key BP genes and processes compared to conventional approaches (Table 2). Additionally, these data indicate that the KDs regulate both the significant BP GWAS genes which likely have stronger influence on pathophysiological outcomes and the genes with more subtle effects which account for the missing heritability. Furthermore, our KD subnetworks retrieved based on a previous BP GWAS meta-analysis with a smaller sample size, was able to predict causal BP genes such as *KIF15, LAMC1, TGFBR2*, and *RBPMS* which hadn't been highlighted until the latest BP GWAS (2) with a larger sample size and power. This strongly supports the accuracy and predictive power of our computational pipeline. These genes are linked to pathways potentially associated with mechanisms modulating blood pressure. Particularly, *LAMC1* coding for Laminin Subunit Gamma 1, has been implicated in the regulation of vascular lumen development (62). Also, proteomic analysis suggests this subunit is involved in ECM remodeling during venous hypertension in varicose veins (63). Furthermore, *TGFBR2* and *RBPMS* show a high degree of expression in smooth muscle cells, the former heavily implicated with predisposition for arterial aneurysms which suggests dysfunctions of the arterial wall, while the latter is involved in regulation of smooth muscle plasticity during development (64, 65).

Moreover, we had a number of suggestive genes arise from our vascular tissue data, highlighted as having specific effects on the ECM including *TIMP2, PSAP, FN1, VCAN*, and *LOX*, each of which have been previously implicated in hypertension (63, 66–68). With the ECM being crucial for maintaining the structural integrity of the vessel wall, it seems intuitive that genes affecting ECM homeostasis may contribute to hypertension. One such gene is *TIMP2*, coding for tissue inhibitor of metalloproteinases 2, which is part of a peptidase family involved in the degradation of the ECM, linked with resistant hypertension and arterial stiffness (66). Additionally, our vascular tissue data suggested genes implicated in smooth muscle contraction such as *CALD1, DUSP5*, and *TAGLN* (69–72).

Despite the comprehensive data integration and discoveries discussed above, there are limitations in our study. First, mapping GWAS SNPs to candidate genes is not straightforward. Chromosome location-based mapping lacks direct evidence for the functions of the reported genes, whereas functional data-supported mapping suffers from incomplete coverage of tissue and lack of power in identifying weak cis-association and trans-regulation. To address these issues, we incorporated different SNP-gene mapping approaches (i.e., chromosomal location vs. eQTLs) and prioritized the tissue-specific pathways shared by SBP and DBP. Second, although we collected eQTL datasets from >40 unique tissues/cells from the GTEx database and other studies, the sample sizes for certain tissues or cells are small, resulting in small numbers of eQTLs for these cells to tissue types to be incorporated in our analysis. This limits the statistical power when used separately and most likely contributes to the lack of significant signals from many of the eQTL sets used. To alleviate this power issue, we pooled the eQTLs from related tissues or cell types in pathway analysis, which may mask certain tissue-specific signals. Third, although our findings provide multiple lines of *in silico* evidence to support the importance of the KDs (e.g., literature mining and BP phenotypes from mouse models), experimental validation of the novel KDs for their roles in regulating the BP GWAS genes, networks, and disease development is necessary in future studies.

Overall, high BP is a major risk factor for cardiovascular disease and has a substantial genetic contribution. Our integrative approach utilizing GWAS, functional genomics, and network modeling revealed a comprehensive system view of biological processes, tissue-specific networks, and regulators that contribute to BP regulation. The common pathways between SBP and DBP are significantly enriched for hypertension/CAD GWAS signals and the top KD subnetworks contain numerous candidate CAD causal genes (Supplementary Table 7). Collectively, these findings provide molecular level mechanistic support for the tight connection between BP and CAD risk. The processes and regulators identified from our study may open new avenues for BP-lowering and CAD therapeutics.

## Author Contributions

YZ researched and analyzed all data and drafted the manuscript. MB and LS participated in data analysis and writing of the manuscript. XS, CL, and IA contributed toward the data analysis. SK, TH, and DL contributed data and assisted with results interpretation. XY supervised study design, data analysis, and manuscript writing. All authors reviewed and edited manuscript.

### Conflict of Interest Statement

The authors declare that the research was conducted in the absence of any commercial or financial relationships that could be construed as a potential conflict of interest.
