# Supplementary material for: Integrative Genomics Analysis Unravels Tissue-Specific Pathways, Networks, and Key Regulators of Blood Pressure Regulation
Source: Front Cardiovasc Med. 2019 Mar 12;6:21. doi: 10.3389/fcvm.2019.00021 (PMC6423920; doi:10.3389/fcvm.2019.00021)
Supplement: Supplementary file 8 [file Data_Sheet_1.PDF]

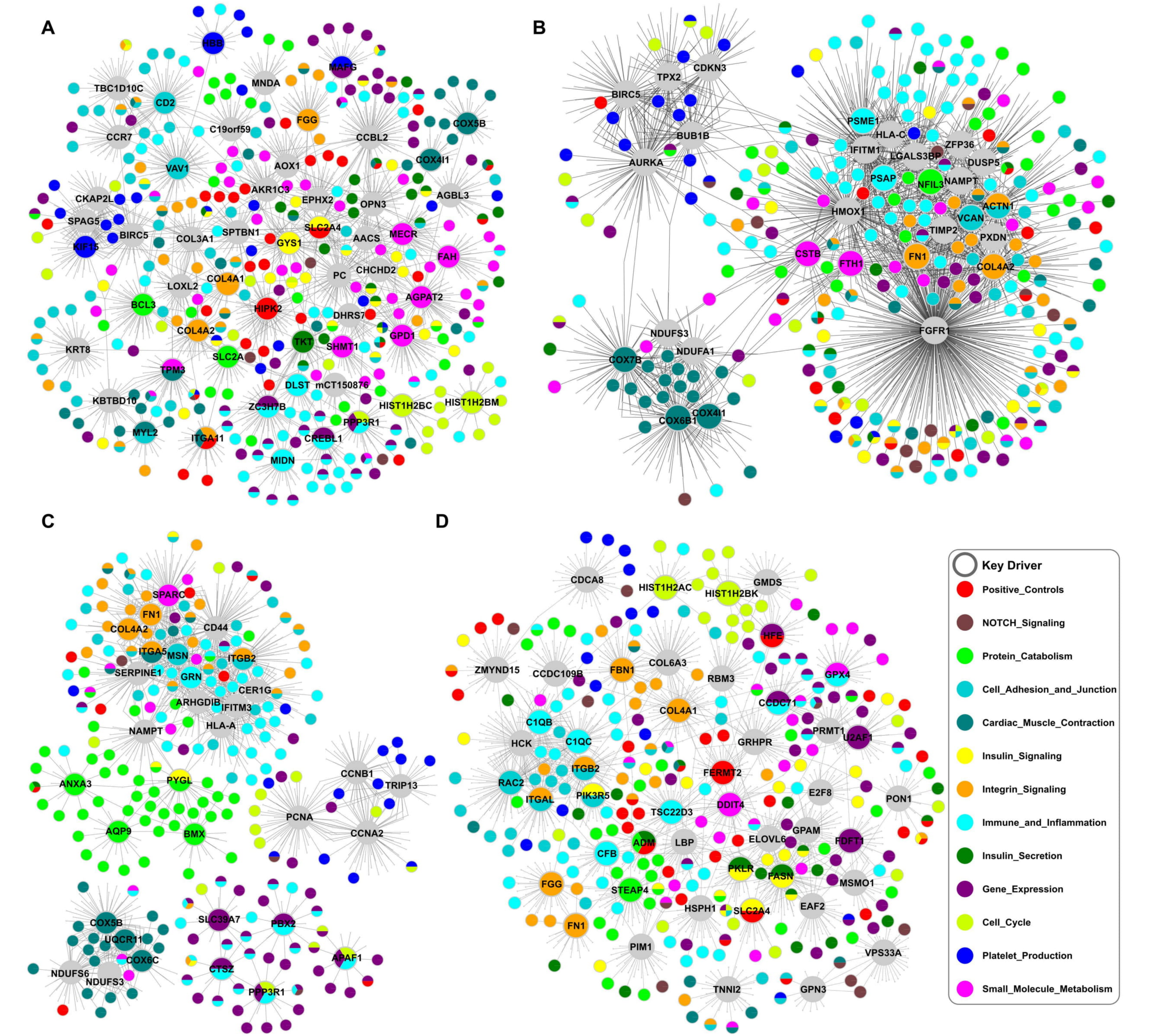

Supplementary Figure 1. The tissue-specific gene regulatory networks of the top KDs of BP. Panel A-D represent networks in adipose, artery, blood, and liver separately. We scaled the nodes according to the following criteria: the largest nodes are key driver genes; the middle-size nodes in color are member genes from the pathways/coexpression modules; and the smallest nodes are nonmember genes that are only slightly wider than their edges. Only the supersets/pathways with >100 genes are illustrated.
